# The predictive utility of unmet need on time to contraceptive adoption: a panel study of non-contracepting Ugandan women

**DOI:** 10.1016/j.conx.2020.100022

**Published:** 2020-03-18

**Authors:** Dana Sarnak, Amy Tsui, Fredrick Makumbi, Simon P.S Kibira, Saifuddin Ahmed

**Affiliations:** a Population, Family and Reproductive Health Department, Johns Hopkins Bloomberg School of Public Health, Baltimore, Maryland, USA; b Epidemiology and Biostatistics Department, School of Public Health, Makerere University, Kampala, Uganda; c Community Health and Behavioral Sciences Department, School of Public Health, Makerere University, Kampala, Uganda

## Abstract

**Objective:**

The predictive utility of the unmet contraceptive need indicator is not well known, despite being recognized as a key family planning indicator for showing the extant demand for birth control. This study assesses the dynamic influence of unmet need on time to contraceptive adoption, as compared with that of contraceptive intentions and their concordance.

**Study design:**

This observational study analyzed survey data, including a contraceptive calendar, reported by a panel of 747 non-contracepting, fecund and sexually active Ugandan women, first interviewed in a 2014 national survey and re-interviewed in 2018. We conducted descriptive, survival and multivariate Cox regression analysis of the influence of women's baseline measures of unmet need, self-reported intention to contracept and their concordance with time to adoption of modern contraception over 36 months.

**Results:**

The study found women classified as having unmet need were slower to adopt contraception than those without unmet need, after adjustment for background covariates (aHR = 0.79, 95% CI = 0.57–1.10). Women intending future contraceptive use were significantly faster to adopt (aHR = 1.45, 95% CI = 1.22–1.73) than those not intending. Women with no unmet need but intending to use had the highest rate of adoption compared to those with no need and no intention to use (aHR = 2.78, 95% CI = 1.48–5.25).

**Conclusions:**

The unmet need indicator underperforms in predicting future contraceptive adoption compared to contraceptive intentions, which merits further consideration as a complementary predictor of future use. Non-contracepting women with unmet need but no intention to use contraception in particular warrant programmatic attention.

**Implications:**

A non-contracepting woman wanting to limit or space her births is defined as having unmet need, but little is known if she subsequently adopts contraception. By contrasting a woman's unmet need with her expressed intention to use, we offer reasons to further consider self-reported contraceptive intentions as a better predictor of adoption and the underlying latent demand for volitional regulation of fertility.

## 1 Introduction

### 1.1 Background

Unmet need for contraception was conceptualized by population scientists in late 1970s and since then has been used as a key family planning indicator for showing the demand for birth control [1]. The construct signals a gap between birth control that women want and what services provide [2]. It is defined as the percent of women of reproductive age (all or married) who want to delay or limit childbearing but are not using contraception. Unmet need levels are globally and annually estimated by the United Nations [3] to track progress toward the 2030 Sustainable Development Goals and used to monitor family planning demand satisfied of target populations [4]. Unmet need for contraception is separately measured for women seeking to space (2 years or more) and to limit births, and satisfaction of unmet need has been identified as potential means for averting maternal deaths [5].

The measurement of unmet need has varied over time with a recent revision [6] standardizing estimates across developing countries. It relies on survey data from women with respect to their fertility preferences (desire to have more children and when), wantedness status of the last pregnancy for women who are currently pregnant or postpartum amenorrheic (then, later, not at all) and current contraceptive use. Women are also asked about their recent sexual activity, infecundity/menopausal, and marital status to gauge their exposure to pregnancy risk, and thereby contraceptive need. Unmet need is then a composite measure defined for non-contracepting women, while met need is defined for contracepting women. Measurement of met need is based on actual contraceptive use levels,.

Unmet need is not a clinical classification or characterization but a behavioral indicator, conceptually framed around a woman's fertility preferences on the assumption that being a non-user with a desire to delay or limit childbearing signals a need for contraception. The measure does not rely on a woman's directly expressed need or desire to use a contraceptive method, her perception of risk to pregnancy, or her intention or interest to use in the future [7]; instead it represents an externally assessed potential need for family planning.

Unmet need is estimated with population-based surveys, primarily in low- and middle-income countries. Because those surveys are largely cross-sectional, there is also limited knowledge of the predictive utility of the unmet need indicator. Little is known about the circumstances by which a woman classified as having unmet need becomes a contraceptive user in the future ([8] Curtis and Westoff, 1996 being an exception). If unmet need status does not predict her future use, its significance as a contraceptive demand indicator may be over-stated. This study addresses the knowledge and evidence gap in the research literature on the predictive utility of the unmet need measure.

### 1.2 Objective

The aim of this study is to assess the predictive validity of the unmet need measure on time to contraceptive adoption using data from a panel of 747 non-contracepting, fecund and sexually active Ugandan women, first interviewed in 2014 and subsequently in 2018. We compare unmet need's influence on her subsequent use with two other measures: her self-reported intention for future use and the concordance between externally defined unmet need and her own intention. Contraceptive intentions may represent a cognitive and necessary step in a woman's decision-making process to use birth control, beyond just her fertility preferences. We thus assess the longitudinal relationship between unmet need, contraceptive intention and their concordance with the probability a woman adopts modern contraceptive method over a three-year period.

## 2 Material and methods

### 2.1 Study design and data

This observational study used survey data collected from the 2014 cross-sectional Performance Monitoring and Accountability (PMA) 2020 Survey for Uganda. PMA2020 surveys (hereinafter referred to as PMA) annually monitor key family planning indicators (see Zimmerman et al., 2017 [9]; www.pmadata.org). In 2014 PMA/Uganda sampled 110 urban and rural clusters and selected 44 households randomly in each cluster, resulting in a national sample of completed interviews for 4295 households and 3800 women of reproductive age (15–49 years). This survey round is hereinafter referred to as Round 1 or R1. In 2018 a second survey (hereinafter R1F) relocated 4146 households of the 2014 sample with 2833 having at least 1 original R1 resident. It then interviewed all resident eligible women (*n* = 2722). Among the female sample, 1716 women were original R1 respondents (45.2%). Those who could not be re-contacted and re-interviewed tended to be unmarried, under age 30, or childless. R1 records could be matched for 1655 women (96.4%).

About two thirds (1137 or 68.7%) were not contracepting at R1. We excluded women not sexually active (257) or menopausal or infecund (114) at R1 to confine the sample to those at risk for future pregnancy. Another 12 women were eliminated due to missing data on several R1 measures, leaving 747 non-contracepting and non-menopausal women in the constructed panel or cohort.

The study's data collection protocols were approved by IRBs at the Makerere University School of Public Health and Uganda National Council for Science and Technology, and at the Bloomberg School of Public Health at Johns Hopkins University.

### 2.2 Methods and measures

The R1F questionnaire measured many of the same items in the R1 questionnaire. One addition was a five-year retrospective reproductive and contraceptive calendar, modeled after the Demographic and Health Survey [15], covering the period June 2013 to June 2018. The calendar data were used to construct our outcome of interest, time to first contraceptive adoption. Time to adoption was measured for each episode of use in months from Round 1, and our observation period was the 36 months after R1. Episodes of use, defined as the duration from baseline to first contraceptive use, were tested both when censored or not censored by a pregnancy. The results were similar; we present the pregnancy-censored findings given the logical sequence of events.

**Fig. 1:**
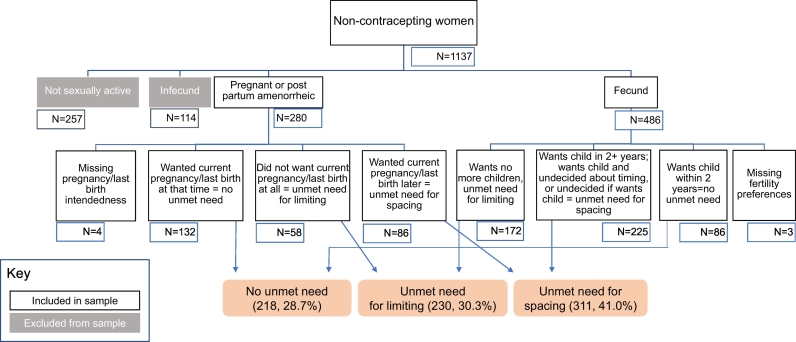
Flow chart for classifying unmet need status of non-contracepting Ugandan female respondents at baseline (2014). Notes: Our unmet need definition is based on Bradley, Sarah E.K., Trevor N. Croft, Joy D. Fishel, and Charles F. Westoff. 2012. *Revising Unmet Need for Family Planning*. DHS Analytical Studies No. 25. Calverton, Maryland, USA: ICF International. Another 12 women were excluded from our analytical sample due to missing data on several R1 measures, leading to the final analytical sample of *n* = 747.

Our three contraceptive need explanatory variables of interest were 1) unmet need, 2) self-reported intention to use contraception, and 3) concordance in unmet need and intention. The R1 and R1F survey questionnaires collected information on most of the components of the current definition of unmet need [6]. The classification scheme had seven categories and its construction is detailed in Fig. 1: Unmet need for spacing, unmet need for limiting, using for spacing, using for limiting, no unmet need, infecund or menopausal, and not sexually active. Non-contracepting women were asked in R1 whether they would use contraceptives in the future, and their responses were classified as: 1) intend to use and 2) do not intend to use. A third category was added of those currently using in R1F to show that some of the non-contracepting women at R1 had adopted by R1F.

The third explanatory variable was an interaction of the first two, “unmet need-intention concordance,” and had four categories: 1) had an unmet need for contraception and intended to use contraception in the future; 2) had an unmet need but did not intend to use contraception; 3) had no unmet need and intended to use; and 4) had no unmet need and had no intention to use.^1^ We hypothesized that non-contracepting women defined externally as having unmet contraceptive need and expressing an intention to use would be the quickest to adopt contraception over a 3-year period.

Women's background characteristics were used as control variables: their age; highest level of schooling; parity; urban–rural residence; marital status and household wealth quintile at R1. Household wealth was based on a score constructed from a principal-components analysis (PCA) of household assets at R1, with the weights applied to the same set of household assets present at R1F. The score distributions were divided into quintiles.

### 2.3 Statistical analysis

We conducted descriptive analyses and used survival analysis methods and multivariate hazard regression models to assess the associations between a woman's unmet need status, her intention to use contraception, and need-intention concordance on time to contraceptive adoption. We constructed Kaplan–Meier survival curves for time to adoption by unmet need status. Log rank tests for statistically significant differences between survival curves were calculated. In the multivariate models, we included the woman's background covariates measured at R1 with the exception of marital status due to collinearity — in the standard unmet need definition, unmarried women are assumed to not be sexually active [6]. The models' standard errors (shown as 95% confidence intervals) were adjusted for clustering given the complex, multi-stage stratified cluster design of the surveys.

## 3 Results

### 3.1 Change in panel participants' composition and outcome

Our panel of non-users in R1 was young (52.6% were under age 30), most had a primary school education (60.6%), were largely married (94.0%) and living in rural areas (90.4%), as seen in column 1 of Table 1. In R1F, we saw the expected shifts in panel composition after 4 years: 67.3% of the cohort was over age 30 and the percent with 5 or more births increased from 40.8 to 58.5% (Column 2, Table 2). The percent of women living in the top 40% wealthiest households also increased from 30.4% to 35.5%.

**Table 1:** Percent distributions of characteristics of panel of non-contracepting exposed Ugandan female respondents at baseline (2014) and follow-up (2018) surveys

	Non-user panel *n* = 747	
Characteristic	At baseline	At follow-up
Total	100.0	100.0
Age (years)		
15–24	28.5	12.2
25–29	24.1	20.6
30–34	19.5	22.5
35–39	15.1	18.1
40–44	8.6	14.6
45 plus	4.2	12.1
Number of live births		
0–2	33.1	14.1
3–4	26.1	27.4
5 or more	40.8	58.5
Schooling level		
None	23.5	21.8
Primary	60.6	62.7
Secondary or higher	16.0	15.5
Wealth quintile		
Lowest quintile	27.9	24.5
Lower quintile	24.0	20.0
Middle quintle	17.8	20.2
Higher quintile	19.9	22.5
Highest quintile	10.5	13.0
Marital status		
Currently married	94.0	90.2
Never married	2.7	2.3
Widowed/divorced/separated	3.4	7.5
Residence location		
Urban	9.6	
Rural	90.4	

In addition to not contracepting, exposed females are sexually active and non-menopausal.

**Table 2:** Percent distributions on fertility and contraceptive behaviors related to unmet need for panel of non-contracepting exposed Ugandan females at baseline (2014) and follow-up (2018) surveys

	Non-user panel *n* = 747	
Characteristic	At baseline	At follow-up
Total	100.0	100.0
Any contraceptive use		
Using a modern method	0.0	25.4
Using a traditional method	–	1.5
Not using	100.0	73.1
Last pregnancy wantedness		
Then	50.9	51.1
Later (mistimed)	25.3	27.0
Not at all (unwanted)	18.9	20.5
Don't know/No answer	0.7	0.0
Never pregnant/given birth	4.3	1.3
Desire for more children		
More children < 2 years	12.7	8.7
More children 2 + years	52.1	42.0
No more children	35.1	45.7
Infertile	–	3.6
No answer	0.1	0.0
Unmet need status		
Unmet need for spacing	40.8	22.4
Unmet need for limiting	30.8	23.2
Using for spacing	–	15.0
Using for limiting	–	11.9
No unmet need	28.4	12.1
Infecund or menopausal	–	9.5
Not sexually active	–	5.9
Type of method adopted by 2018 (*n* = 249)		
Female sterilization	–	7.5
Male sterilization	–	0.0
Implants	–	23.4
IUD	–	3.0
Injectables	–	46.8
Pill	–	6.0
Male/female condoms	–	5.5
Emergency contraception	–	0.5
Other modern (Diaphragm, beads, LAM)	–	1.5
Rhythm	–	3.0
Withdrawal	–	2.5
Intention to use modern contraception in future		
Yes	58.2	39.3
No	41.8	18.4
Currently using	–	26.9
Infecund or menopausal	–	9.5
Not sexually active	–	5.9

In addition to not contracepting, exposed females are sexually active and non-menopausal.

Table 2 shows change in contraceptive prevalence, fertility preferences, pregnancy wantedness, and the contraceptive need measures of interest. In R1, none of the cohort was using contraception; and in R1F 25.4% of women were using modern methods. Of them, close to half were using injectables and one third long-acting methods (female sterilization, implants, or IUD), with the remainder using condoms, pills, emergency contraception or other methods. In R1 44.2% of women's last pregnancies were reported as mistimed or unwanted, with the level slightly higher at 47.5% in R1F. The panel's fertility preferences changed over time — those wanting to postpone two or more years declined from 52.1 to 42.0% and those wanting no more births increased from 35.1% to 45.7%.

Unmet need distributions shifted over the two surveys, both as a result of women adopting contraception, as well as due to changes in sexual activity and infecundity. While 40.8% of R1 women had an unmet need for spacing, 22.4% did in R1F. Unmet need for limiting declined from 30.8% to 23.2%. About one quarter (28.4%) of women was categorized as having no unmet need in R1, dropping to 12.1% in R1F. Over half of R1 respondents (58.2%) reported an intention to use and 41.8% reported no intention, with R1F percentages reducing to 39.0 and 18.4% respectively, as 26.9% were contracepting.

### 3.2 Main results

**Fig. 2:**
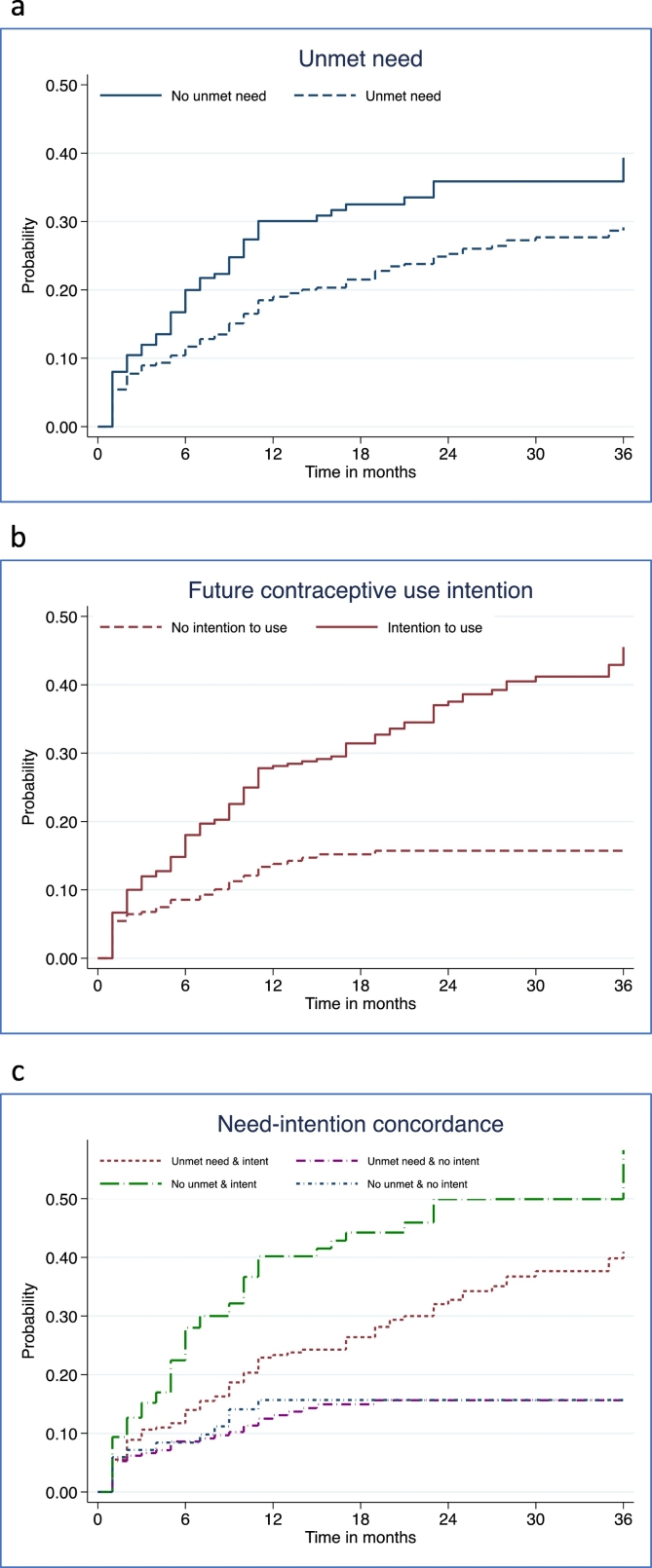
Cumulative proportion of non-contracepting exposed Ugandan women adopting modern contraception over 36 months by baseline unmet need (a), intention (b) and need-intention status (c).

Table 3 and Fig. 2a–c show contraceptive adoption rates over the 36-month observation period from the Kaplan-Meir survival analysis by their unmet need status (2a), intention to use (2b), and need-intention concordance (2c). About two-fifths of R1 respondents (307/747 or 41.1%) were categorized as concordant on unmet need and intention to use in the future, 30.5% (307/747) had unmet need but no intention to use in the future, and 11.2% (84/747) had no unmet need and no intention to use.

**Table 3:** Cumulative proportion adopting modern contraception by selected intervals and baseline unmet need and intention status among Ugandan females⁎

		Cumulative percent adopting by month						
Unmet need/Intention status	N	6 months	12 months	18 months	24 months	30 months	36 months	Significance level
Unmet need status	747							p <.01
No unmet need	212	20.0	30.1	32.5	35.9	35.9	39.3	
Unmet need	535	11.7	19.0	21.5	25.3	27.7	29.1	
Intention to use	747							
No intention to use	312	8.6	13.8	15.2	15.7	15.7	15.7	p <.01
Intention to use	435	18.0	28.1	31.4	37.5	41.2	45.5	
Unmet need and intention concordance	747							
Unmet need and intention to use	307	14.0	23.4	26.4	32.8	37.7	41.0	p <.01
Unmet need and no intention to use	228	8.6	13.1	15.0	15.7	15.7	15.7	
No unmet need and intention to use	128	28.0	40.2	44.2	49.9	49.9⁎⁎	58.3⁎⁎	
No unmet need and no intention to use	84	8.4	15.7	15.7	15.7	15.7	15.7	

Significant differences between survival curves based on log rank test.

⁎ Non-contracepting, sexually active and non-menopausal female participants at 2014 baseline survey.

⁎⁎ Based on less than 20 cases.

Unexpectedly, more women with no unmet need in R1 adopted by 36 months, compared to women with unmet need (39.3 versus 29.1%, see Fig. 2a). Women who intended to use adopted more rapidly than those who did not intend across the 3-year period; Fig. 2b also shows they adopted more quickly by each interval. In terms of need concordance, fastest to adopt were those discordant with no unmet need but an intention to use (see Fig. 2c), with nearly half (49.9%) adopting by 24 months, as compared to women with concordance in unmet need and intention to use (32.8%). Women who did not intend to use, whether classified as having or not having unmet need, were the least likely to adopt, leveling off at 15.7% by 24 months.

Table 4 presents the unadjusted and adjusted results from the hazard regression models for each contraceptive need indicator's association with time to adoption. The unadjusted hazard ratio (HR) was 0.66 (95% CI = 0.48–0.89) for unmet need and the ratio adjusted for background covariates was 0.79 (95% CI = 0.57–1.10). The unadjusted and adjusted HRs for contraceptive intention were 1.65 (95% CI = 1.39–1.96) and 1.45 (95% CI = 1.22–1.73) respectively. The concordance category with the highest predictive value was ‘no unmet need-intend to use’ (aHR = 2.78, 95% CI = 1.48–5.25), followed by ‘unmet need-intend to use’ (aHR = 1.98, 95% CI = 1.07–3.66), where the reference group was ‘no unmet need-no intention to use.’ The overlap in the confidence intervals suggests that intention for future use was the key predictor, irrespective of unmet need status.

**Table 4:** Results from Cox hazard regressions of time to contraceptive adoption by baseline unmet need, contraceptive intention and concordance measures, unadjusted and adjusted for background covariates: Panel of non-contracepting exposed Ugandan females (*n* = 747).

Baseline unmet need, intention to use and background covariate	Hazard ratios and 95%CI		Hazard ratios and 95%CI		Hazard ratios and 95%CI	
	Unadjusted	Adjusted	Unadjusted	Adjusted	Unadjusted	Adjusted
N	747	742	747	742	747	742
Unmet need status						
No unmet need	Ref	Ref				
Unmet need	**0.66 (0.48–0.89)**	0.79 (0.57–1.10)				
Contraception Intentions						
No intention to use			Ref	Ref		
Intention to use			**1.65 (1.39–1.96)**	**1.45 (1.22–1.73)**		
Unmet need concordance						
No unmet need-no intention to use					Ref	Ref
Unmet need-intention to use					**2.16 (1.18–3.95)**	**1.98 (1.07–3.66)**
Unmet need-no intention to use					0.91 (0.47–1.77)	1.06 (0.54–2.09)
No unmet need-intention to use					**3.65 (1.95–6.85)**	**2.78 (1.48–5.25)**
Age						
< 25 years		Ref		Ref		Ref
25–34 years		**0.66 (0.44–0.99)**		0.70 (0.47–1.04)		0.67 (0.45–1.01)
35 + years		**0.32 (0.18–0.56)**		**0.39 (0.22–0.71)**		**0.39 (0.22–0.70)**
Parity						
0–2 children		Ref		Ref		
3–4 children		1.26 (0.84–1.90)		1.16 (0.78–1.72)		1.23 (0.82–1.85)
5 + children		1.32 (0.80–2.20)		1.13 (0.69–1.87)		1.24 (0.74–2.08)
Education						
Never attended		Ref		Ref		Ref
Primary		**2.27 (1.37–3.76)**		**2.00 (1.20–3.32)**		**1.97 (1.19–3.29)**
Secondary or higher		**3.27 (1.79–5.89)**		**2.77 (1.52–5.05)**		**2.74 (1.49–5.02)**
Residence						
Urban		Ref		Ref		Ref
Rural		0.73 (0.45–1.18)		0.75 (0.46–1.21)		0.74 (0.46–1.20)
Wealth Quintile						
Lowest quintile		Ref		Ref		Ref
Lower quintile		1.18 (0.76–1.84)		1.10 (0.71–1.71)		1.07 (0.69–1.67)
Middle quintile		1.25 (0.77–2.04)		1.20 (0.74–1.95)		1.19 (0.73–1.93)
Higher quintile		1.34 (0.85–2.10)		1.28 (0.81–2.01)		1.26 (0.80–1.98)
Highest quintile		1.13 (0.63–2.03)		1.17 (0.65–2.10)		1.11 (0.61–2.00)

Marital status not included due to collinear definition of unmarried with not sexually active in unmet need.

Boldfaced hazard ratios indicate statistical significance at p <.05 or better.

In addition to not contracepting, exposed females are sexually active and non-menopausal.

Women over age 35, compared to those under 25, were significantly slower to adopt, while women with any education, as compared to with none, were significantly faster to adopt contraception. Baseline covariates of parity, urban residence and household wealth were associated with higher adoption rates, although their HRs were not statistically significant. We estimated a model using time to adoption of a short-acting method as the outcome and obtained similar results (data not shown).

### 3.3 Other analyses

We investigated the composition of the samples in the four concordance groups (Appendix Table A1) and observed that classifying women as having no unmet need on the basis of having desired births recently or wanting another birth soon removed many who would go on to adopt contraception for spacing purposes. We further explored the reasons women reported for not using contraception in Round 1^2^ by unmet need status and observed that women classified as having an unmet need and an intention to use in the future were more likely to cite reasons related to limited exposure to pregnancy risk, such as breastfeeding and no menses since birth, compared to their counterparts with unmet need and no intention to use. Those with unmet need and no intention to use were more likely to report fear of side effects and health concerns, as well as opposition to contraception as reasons for non-use.

## 4 Discussion

### 4.1 Key results

Despite wide global reliance on the measure as a key family planning indicator, the predictive validity of contraceptive unmet need is not well established. Our panel study of non-contracepting, sexually active and fecund Ugandan women has shown that baseline unmet need is not a strong predictor of subsequent contraceptive adoption. Many women traditionally classified as having no-unmet need adopt a contraceptive method at a much higher rate than expected as their own perceived risk and need may be different. We find women expressing a future intention to use have 1.45 times faster the adoption rate than the rate of women with no intention. Irrespective of their unmet need status, women with an intention to use adopted nearly two times faster than those with no intention.

The definition of unmet need is static and can underestimate demand from those with recent and future desired pregnancies since fertility preferences will change with time. In addition, unmet need's underlying constructs of fertility preferences and wantedness of last pregnancy are applied to non-contracepting females but do not account for psychosocial costs they associate with contraception, including interest in or resistance to contraception, which are different from fertility-related desires. A recent follow-up study of women in Ghana found that many of the measures on which unmet need is based were shown to be unreliable even over a short period of time, including fertility preferences, sexual activity and discrepancies in method use [[1], [7]], which may explain why the predictive utility of unmet need on contraceptive adoption is weak in our study. Constructing alternative measures of contraceptive demand, such as based on self-reported intentions to use contraception, that provide greater predictive value than unmet need, merits consideration. Self-reported contraceptive intention reflects women's personal recognition of family planning as a relevant means to manage their childbearing.

### 4.2 Strengths and limitations

Our panel sample is drawn from less than half of the original sample but is composed of non-contracepting women who at baseline are exposed to pregnancy risk and who are primarily in union, rural, in poor households and at high parity, the population type that contraceptive outreach programs are designed to address. Being able to observe this panel's reproductive behaviors over a 36-month period has allowed us to compare the different trajectories of contraceptive uptake for defined need categories and assess the persistent strength of women's baseline intentions to use, while expanding their families.

### 4.3 Interpretation

Unmet need's programmatic value is the assessment of the gap between proportions of the female population with met and unmet need, guiding program service response to enable more couples to access and use contraception. The unmet need construct, however, is nuanced and warrants an improved understanding of underlying psychosocial dynamics, such as ideational change around fertility preferences, while contraceptive intentions merits independent research on its ideational formation [10,11,12]. A complementary focus on contraceptive intentions as a separate and proximal predictor of future use may help reduce unmet need. Service barriers, such as contraceptive commodity stock-outs and judgmental clinical providers [13,14], will also contribute to unmet need. Concerns about contraceptive side effects and health impacts are frequently reported and point to need for improved provider counseling. Our results suggest family planning programs should focus a greater share of their efforts understanding and addressing barriers faced by women with unmet need and no intention to use, as this group appears to be the least likely to seek out and use services, despite stated desires to space or limit childbearing.

## Funding

This study received support from the Bill & Melinda Gates Foundation, Seattle, WA [grant numbers 1079004 and 1163880].

## Footnotes

1 Four of the seven baseline unmet need categories did not apply in constructing the interaction term: using for spacing or limiting, being menopausal or infecund or not sexually active.

2 Reasons for non-use is only asked among women desiring to space or limit future childbearing and therefore this sub-analysis does not include the entire sample
